# Fabrication of phase masks from amorphous carbon thin films for electron-beam shaping

**DOI:** 10.3762/bjnano.10.128

**Published:** 2019-06-25

**Authors:** Lukas Grünewald, Dagmar Gerthsen, Simon Hettler

**Affiliations:** 1 Laboratory for Electron Microscopy, Karlsruhe Institute of Technology (KIT), Engesserstrasse 7, 76131 Karlsruhe, Germany

**Keywords:** amorphous carbon, Bessel beam, electron-beam shaping, nanofabrication, vortex beam

## Abstract

**Background:** Electron-beam shaping opens up the possibility for novel imaging techniques in scanning (transmission) electron microscopy (S(T)EM). Phase-modulating thin-film devices (phase masks) made of amorphous silicon nitride are commonly used to generate a wide range of different beam shapes. An additional conductive layer on such a device is required to avoid charging under electron-beam irradiation, which induces unwanted scattering events.

**Results:** Phase masks of conductive amorphous carbon (aC) were successfully fabricated with optical lithography and focused ion beam milling. Analysis by TEM shows the successful generation of Bessel and vortex beams. No charging or degradation of the aC phase masks was observed.

**Conclusion:** Amorphous carbon can be used as an alternative to silicon nitride for phase masks at the expense of a more complex fabrication process. The quality of arbitrary beam shapes could benefit from the application of phase masks made of amorphous C.

## Introduction

The possibility to shape electron beams has gained much interest since the first observation of electron vortex beams, i.e., beams that carry a defined orbital angular momentum [1–3]. Various other beam shapes, e.g., non-diffracting Bessel beams [4–7] or Airy beams [8–10], were realized soon after. The special properties of these beam shapes can be used in scanning (transmission) electron microscopes (S(T)EMs) to obtain more information about a sample. For example, electron vortex beams can be applied to study magnetic states in ferromagnetic materials [3]. The non-diffracting behavior of Bessel beams could be used as an electron probe with enhanced depth of focus [4] as for conventional (sub-angstrom) electron probes the depth of focus is reduced to a few nanometers due to the large convergence angles used in modern (aberration-corrected) microscopes [11].

A range of different techniques has been developed to experimentally realize electron-beam shaping [12]. For example, special slit apertures [6–7] or nanoscale amplitude holograms [3] block specific parts of the incoming electron wave and generate the desired beam shape below the structure. However, a main drawback of these amplitude-modulating techniques is the relatively large intensity loss in the beam-generation process. Hence, other techniques use a modulation of the phase instead of the amplitude. For example, a magnetic tip [13] or a (detuned) aberration corrector [14] are possible ways to alter the phase. In our work we choose two more commonly used phase-modulating approaches in the form of refractive and holographic phase masks to generate Bessel and vortex beams.

In both methods, the required phase-shift difference between different regions of an incoming electron plane wave is generated by an amorphous thin film with locally varying thickness. In general, electrons undergo a thickness-dependent phase shift 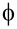 in a thin, amorphous, non-magnetic material according to [15]:

[1] $$\phi \left(x,y\right)=C_{\text{E}}V_{\text{MIP}}t\left(x,y\right)$$

Here *C*_E_ denotes the energy-dependent interaction constant (6.53 × 10^−3^ rad·nm^−1^·V^−1^ for a primary electron energy of *E* = 300 keV), *V*_MIP_ is the mean inner potential (MIP) of the material, *t* is the thickness of the thin film and *x* and *y* are the directions perpendicular to the incident electron beam. The underlying effect is analogous to the phase shift that is generated between light rays traversing media with different refractive indices. As an example, 300 keV electrons acquire a phase shift of π in a 53 nm thick amorphous carbon film (*V*_MIP_ = 9 V [16]). Due to the small film thickness needed for phase shifts of the order of π, most electrons propagate through the structure without any (in-)elastic scattering events, i.e., the amplitude is only modified slightly.

Experimentally, focused ion beam (FIB) milling or electron-beam lithography are used to engrave a well-defined thickness profile in an amorphous thin film thereby exploiting the direct proportionality between 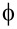 and *t*. The structured film is surrounded by an obstructing aperture with a diameter of a few (ten) micrometers to block (or scatter) electrons that do not hit the patterned thin film. Such a device is called a phase mask (PM). A distinction is made between refractive and holographic PMs, which differ in the design of the thickness pattern. Refractive PMs directly mimic the required phase shift with the thickness pattern, e.g., the helical phase shift required for the generation of vortex beams is realized by a helical thickness ramp [17]. In the holographic approach the thickness pattern is calculated based on a superposition of the desired target wave ψ_target_ and a reference wave ψ_ref_, i.e., 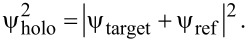 A tilted plane wave is commonly taken as ψ_ref_, although other wave types can also be used [18–19]. Refractive PMs have the ability to generate a single beam with high intensity whereas the holographic approach produces multiple diffracted beams and the intensity is distributed between them.

Silicon nitride (Si*_x_*N*_y_*) has been exclusively used as an amorphous material for PMs up to now. It is characterized by high mechanical robustness and low scattering probability for electrons. As a practical aspect, smooth, free-standing Si*_x_*N*_y_* thin films are commercially available. Smooth thin films are a requirement for the successful fabrication of the thickness pattern. However, Si*_x_*N*_y_* is an insulator and an additional conductive layer has to be deposited onto a Si*_x_*N*_y_*-based PM to avoid charging by electron-beam irradiation, which in turn increases scattering.

In this work we investigated amorphous carbon (aC) as an alternative PM material. Amorphous carbon, like Si*_x_*N*_y_*, offers high mechanical stability, low scattering probability and in addition high electrical conductivity. Because of these properties, aC is commonly used in other phase-related techniques in the form of phase plates, e.g., Zernike phase plates in phase-plate TEM [20]. In the latter application, effects such as contamination, beam damage and charging of aC phase plates due to intense electron-beam irradiation in the back focal plane of the objective lens are known problems. These effects are expected to only marginally affect the PM performance because of the almost parallel illumination of the PMs leading to a substantially reduced areal electron dose [17,21]. We have developed two different procedures to fabricate aC PMs in this work. The properties of the PMs were evaluated by implementing them in the object plane of a transmission electron microscope. Finally, electron vortex and Bessel beams were successfully generated by installing PMs in the condenser lens system.

## Theoretical Background

### Bessel beams

Bessel beams (BBs) are named after their transverse beam profile in form of a squared Bessel function of the first kind and *m*-th order, 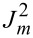, which were first discussed and experimentally realized in light optics [22–23]. For *m* = 0 the global maximum of the Bessel function lies at the origin, making this case interesting for an electron probe as the global maximum is then located on the optical axis (on-axis BBs). In theory [22], the shape of BBs does not change upon propagation, hence they are denoted as non-diffractive beams. Experimentally, BBs possess their non-diffractive property only for a certain propagation distance *z*_max_ due to finite aperture sizes [4,23–24]. The value of *z*_max_ can be estimated by

[2] $$z_{\text{max}}\approx \frac{D}{2\lambda k_{\rho }}\;,$$

where *D* is the diameter of the PM, λ the relativistic electron wavelength and *k*_ρ_ the magnitude of the wave vector in the PM plane. The latter is defined by 
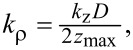
 according to the geometrical relationship between the magnitude of the wave vectors *k*_ρ_ and *k*_z_ and their real space equivalents *D*/2 and *z*_max_, respectively (see also Figure 4 in [25]). Equation 2 then follows from the approximation *k* = 1/λ ≈ *k*_z_ with the wave vector 
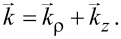
The value of *k*_ρ_ is an important experimental parameter because it determines the shape of the generated BB. If the argument of the Bessel function is given by *k*_ρ_ρ with the radius ρ = √(*x*^2^ + *y*^2^), the diameter of the central lobe of 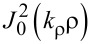 scales inversely with *k*_ρ_. Consequently, a larger *k*_ρ_ value results in a smaller central maximum and therefore potentially in a finer electron probe. However, an increase in *k*_ρ_ with constant *D* also increases the number of side maxima, which could lead to unwanted signal from other sample areas contributing to the main signal in a STEM experiment. Furthermore, each ring of a BB contains roughly the same intensity, which means that additional rings reduce the intensity in the central lobe [26]. In practice, this may result in a reduced signal-to-noise ratio and a compromise has to be found between a large *k*_ρ_ and sufficient intensity in the central lobe [25].

While the radial beam profile in form of a Bessel function is preserved upon propagation up to *z*_max_, the intensity distribution between the rings changes. In particular, the intensity of the central lobe on the optical axis shows a linear increase with superimposed oscillations [5,24]. This feature is used in this work to identify BBs by tracing the intensity of their central lobe upon propagation.

To generate a single BB with a radial profile of the form 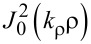 on the optical axis of a transmission electron microscope, we opted for a refractive PM with a thickness profile of the form [24]:

[3] $$t_{\text{Bessel}}\left(\rho \right)=t_{0}+\frac{t_{\text{a}}}{2}\left(1-\cos\left(2\pi k_{\rho }\rho \right)\right)\;$$

The parameters *t*_a_ and *t*_0_ describe the thickness amplitude of the sinusoidal structure (*t*_a_) and a remaining offset thickness of the thin film (*t*_0_). Equation 3 corresponds to a pattern of concentric rings with a spacing of 1/*k*_ρ_, which also means that the parameter *k*_ρ_ can be adjusted in the fabrication process. Experimentally, *k*_ρ_ is ultimately limited by the resolution of the fabrication method.

### Vortex beams

Vortex beams (VBs) are of great interest due to their well-defined orbital angular momentum (OAM) 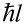 with the topological charge *l* and the Dirac constant 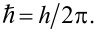 The phase of a VB varies azimuthally upon propagation, where *l* is equal to the number of turns in the wave front per wavelength [27]. In the center of a VB exists a phase singularity (“vortex”) which leads to local destructive interference and the characteristic doughnut-shaped beam profile. In contrast to refractive PMs for the generation of BBs, we alternatively chose the holographic approach to generate off-axis VBs in a TEM. “Off-axis” means that multiple, spatially separated beams are produced by the hologram. Additionally, each of these beams carries a specific amount of OAM, which allows one to select an electron beam with specific OAM if the beams are separated sufficiently. Thickness patterns for the PMs given by

[4] $$t_{\text{Vortex}\text{,cos}}\left(x,\theta \right)=t_{0}+\frac{t_{\text{a}}}{2}\left(1-\cos\left(l_{0}\theta +2\pi fx\right)\right)$$

and

[5] $$t_{\text{Vortex}\text{,saw-tooth}}\left(x,\theta \right)=t_{0}+\frac{t_{\text{a}}}{2\pi }\left(\pi -\mathrm{mod}\left(l_{0}\theta +2\pi fx,2\pi \right)\right)\;$$

were investigated [28]. Here, the variable θ denotes the azimuthal angle, *l*_0_ describes the order of the fork-like structure in the center of the PM and *f* is the spatial frequency of the hologram grating in the *x*-direction, which is used to separate the beams with different OAM. A larger *f* value leads to a stronger separation of the diffraction orders. The sinusoidal phase profile from Equation 4 gives rise to a symmetrical intensity spread between the diffraction orders. This is not optimal if only one specific OAM value is desired for an experiment because much of the incoming intensity on the PM is distributed in other beam orders with unwanted OAM. This issue can be partially overcome by a saw-tooth-shaped phase profile (Equation 5). This way, an intensity asymmetry is produced which can be exploited to generate an intense diffraction order with the desired OAM [28–29].

## Results and Discussion

### Fabrication of amorphous carbon phase masks

#### Fabrication of Si*_x_*N*_y_* membranes and aC film deposition

**Figure 1:**
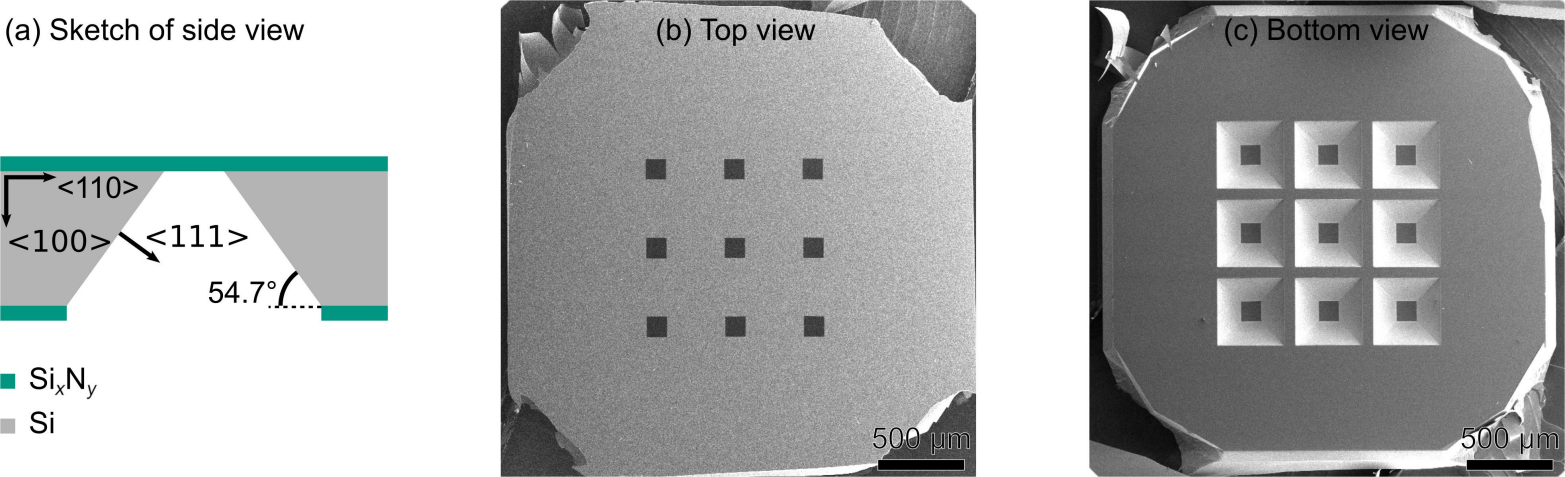
Samples after optical lithography and etching. (a) Scheme of side view of a single membrane. The sketch is not to scale as the Si*_x_*N*_y_* windows and the Si wafer have a thickness of 120 nm and 200 µm, respectively. SEM images of the (b) top and (c) bottom surface reveal the 3 × 3 array of Si*_x_*N*_y_* membranes and the pyramid-shaped trenches caused by anisotropic wet-etching of Si.

**Figure 2:**
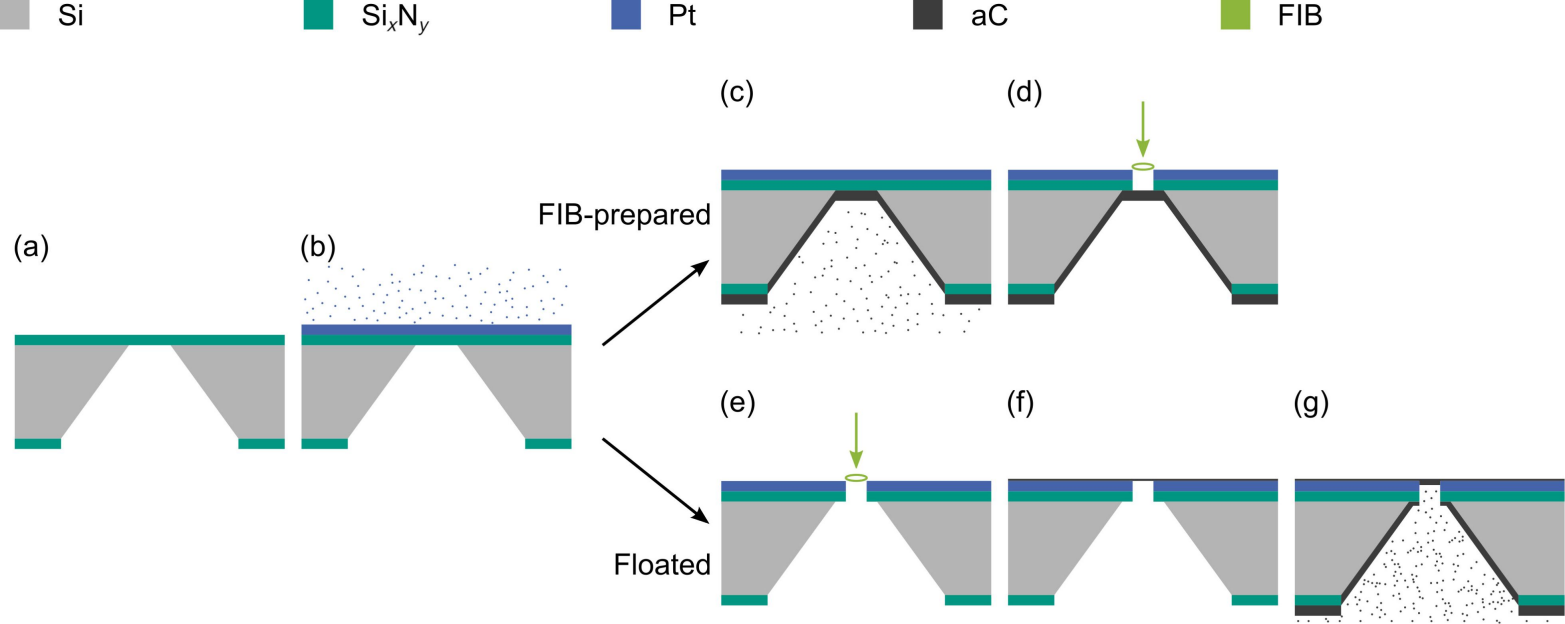
The fabrication steps to fabricate aC thin films are schematically shown for the two applied methods. (a) Si*_x_*N*_y_* membranes prepared by optical lithography are (b) covered with Pt (ca. 220 nm). (c) For the “FIB-prepared” method, aC is deposited on the back side and (d) FIB milling is used to remove Pt and Si*_x_*N*_y_*. The milling is stopped at the homogeneous aC layer. (e) Alternatively, for the “Floated” method, apertures are first created by FIB milling. (f) Amorphous carbon thin films are then floated onto the samples. (g) The thickness of the aC thin films is increased by evaporation of aC.

A 200 µm thick Si wafer with 120 nm thick low-stress Si*_x_*N*_y_* coating on both sides was used as base material. Optical lithography and etching methods for Si*_x_*N*_y_* and Si were applied to produce free-standing Si*_x_*N*_y_* thin films (Figure 1). Pyramid-shaped trenches are generated beneath the thin films by anisotropic wet-etching of Si in a heated KOH solution (KOH + H_2_O in a ratio of 2:3, 80 °C, Figure 1a,c). The lithography mask was designed such that a 3 × 3 array of square-shaped Si*_x_*N*_y_* thin films was produced on a single wafer (Figure 1b,c), which has a diameter of ca. 3 mm to be mounted later in a TEM sample or aperture holder. Ideally, this design makes it possible for a microscope operator to choose from nine different PMs with different beam shapes depending on the experimental needs. Alternatively, such wafers can also be bought directly (Plano, Art. No. 21529-10). To create an electron-blocking aperture, a 3.5 nm/220 nm thick Cr/Pt layer was deposited onto the Si*_x_*N*_y_* via PVD (Figure 2b, Cr not shown) with Cr acting as an adhesion layer.

**Figure 3:**
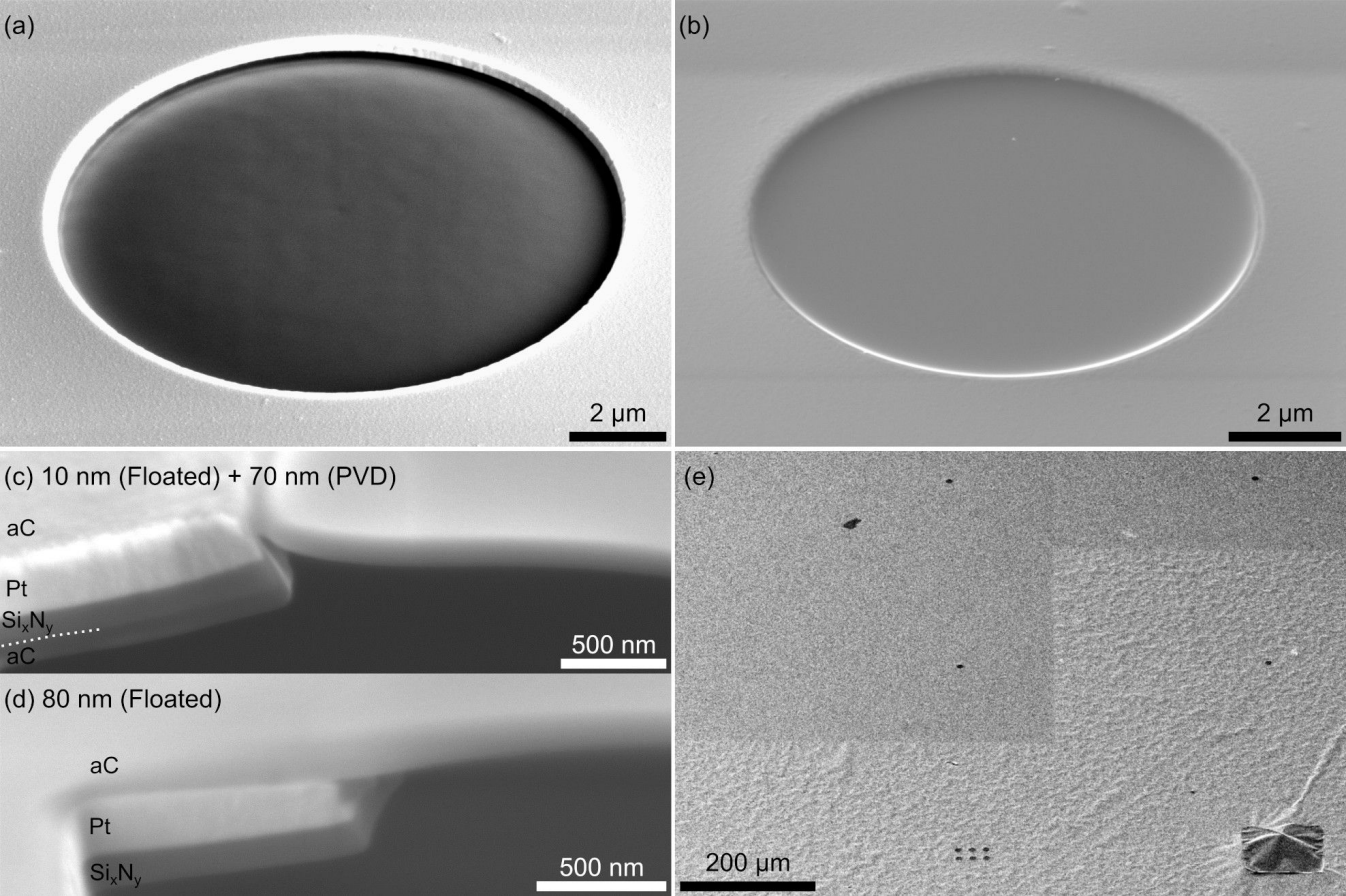
SEM images of (a) FIB-prepared and (b) floated aC thin films reveal a smoother surface for the latter. (c) The cross-section SEM image at the aperture edges reveals sagging of a floated thin film (10 nm) after additional deposition of 70 nm aC. (d) Floating of a comparably thick 80 nm aC film results in more stability at the aperture edge. (e) Delamination of floated aC films is visible in the bottom half of the SEM image. FIB scanning over relatively large areas flattens the film, which is visible in the top part. The straight edges of the FIB scanning windows are clearly visible.

To achieve smooth aC thin films in combination with a circular Pt aperture, we have applied two different approaches that yielded reproducible results (Figure 2). For the first method an aC layer was evaporated on the back side of the wafer by PVD (Figure 2c). Afterwards, FIB milling with an intermediate current of 0.75 nA was used to remove Pt and Si*_x_*N*_y_* in a circular area from the top side (Figure 2d). We used circle diameters (aperture sizes) of 10 µm and 20 µm. Continuous scanning electron microscopy (SEM) imaging with a secondary-electron detector was employed during milling to observe the transition between different material layers. FIB milling was stopped when a homogeneous contrast after the transition between Si*_x_*N*_y_* and aC was observable. This procedure leaves a free-standing aC thin film with slight inhomogeneities (Figure 3a), which are attributed to an inhomogeneous, grain-orientation-dependent sputter rate of the nanocrystalline Pt due to ion-channeling effects [30].

The second method uses floating of a thin aC film (Figure 2e–g). The floating technique is commonly applied to deposit thin aC support films on TEM grids. For thin film preparation, aC with different thicknesses of 10 nm, 30 nm and 80 nm was evaporated onto freshly cleaved mica sheets by PVD. With FIB milling at intermediate to high currents (0.9 to 2.4 nA, depending on the diameter of the aperture) Pt and Si*_x_*N*_y_* were removed in a circular pattern in each of the Si*_x_*N*_y_* windows to create apertures (Figure 2e). Afterwards, the prepared aC films from the mica sheets were floated down onto the Pt side of the samples in a distilled water bath so that the aC films cover the apertures (Figure 2f). After drying for a few days at room temperature in air, additional aC was evaporated by PVD onto the Si*_x_*N*_y_* side to increase the thickness of the aC thin films up to the desired thickness (Figure 2g). The resulting thin films were considerably smoother compared to the technique described first (cf. Figure 3a,b). However, we observed delamination of the aC film from the Pt layer. This effect was more pronounced for thicker aC films, e.g., an 80 nm thick film was detached from the Pt layer when it came in contact with another surface, such as a Kimtech wipe (Kimberly-Clark Professional). Thinner films showed better adhesion although sagging of these films on the aperture edges was observed, which was less pronounced for thicker films (cf. Figure 3c,d). As a compromise, an intermediate thickness of 30 nm for the floated aC films was used. We noticed that a partially delaminated aC film can be flattened by scanning the FIB in a relatively large scanning window (in our case 708 µm × 472 µm) over the delaminated film for around 10 s, making it possible to recover these samples (Figure 3e). For this process a current of 1.2 nA, 3072 × 2048 pixels and a dwell time of 50 ns was used which results in a dose of around 0.2 ions/nm^2^.

#### Phase-mask patterning

In the case of BBs the required thickness profile (Equation 3) was milled with custom FIB routines. These are realized in the form of text files in which the spatial coordinates for the FIB are listed chronologically with their respective dwell times (“stream files”). We aimed for relatively large *k*_ρ_ values in the range of 5 to 10 µm^−1^ resulting in a spacing of 200 to 100 nm between the concentric rings. This choice was made with the aim of decreasing the central peak size of the BB and to test the resolution limits of FIB milling for structuring aC.

**Figure 4:**
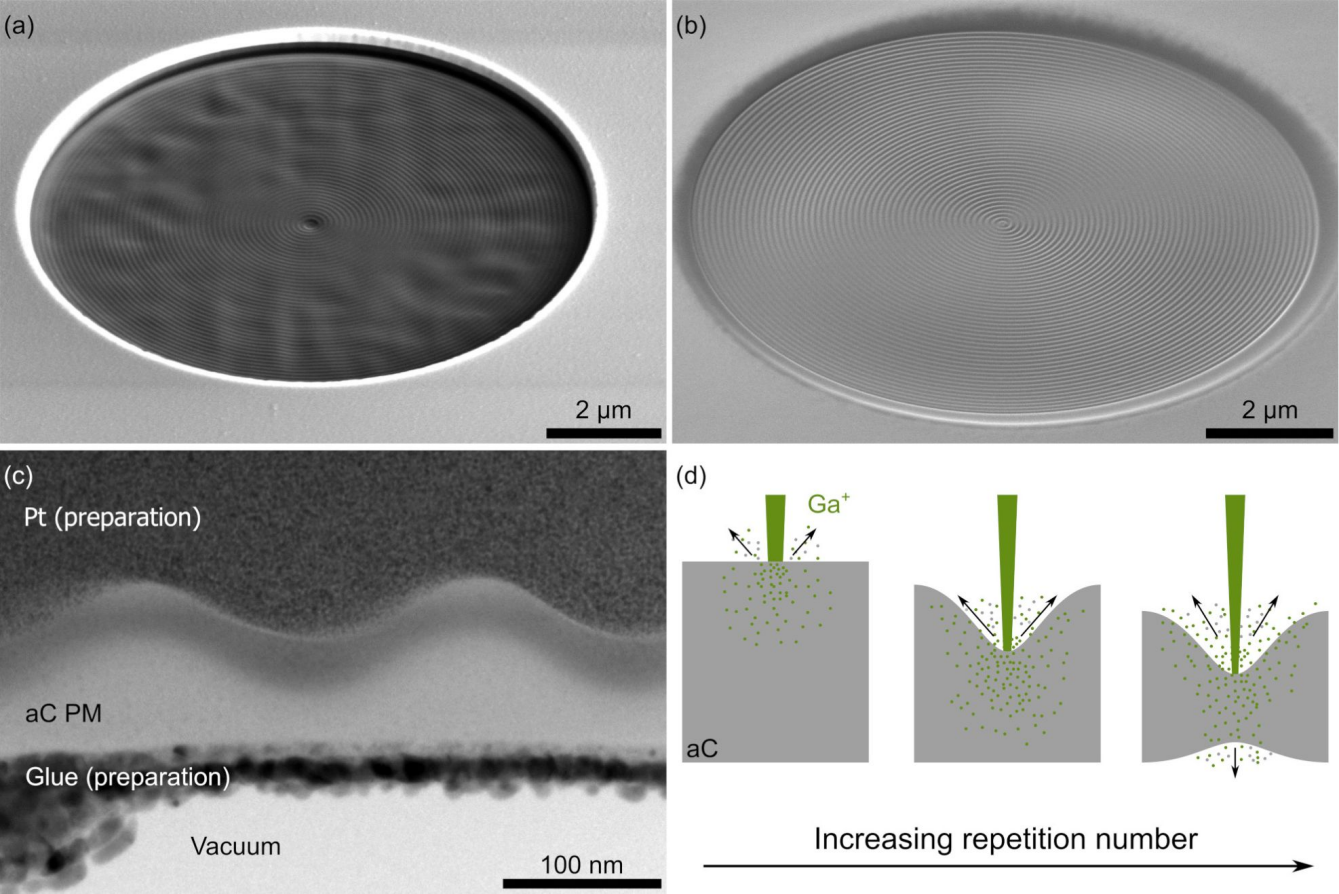
SEM images after structuring the pattern according to Equation 3 with *k*_ρ_ = 10 µm^−1^ for (a) a FIB-prepared and (b) a floated aC thin film. Since the latter is smoother, the sinusoidal structure has a better quality. (c) The generated sinusoidal structure is visible in the bright-field TEM image of a cross-section lamella of a PM with *k*_ρ_ = 5 µm^−1^. Pt was deposited on top to protect the structure during TEM lamella fabrication. We stabilized the free-standing thin films for the lamella preparation by filling the pyramid-shaped trenches with glue. (d) The scheme presents a possible explanation for fast hole formation towards the end of the pattern milling process. The middle scheme corresponds to the situation shown in (c).

To generate a smooth, sinusoidal pattern in radial direction, we opted to drive the FIB only in the minima of the sinusoidal profile. In this routine, the FIB starts in the middle, moves radially outward to the first concentric ring and then mills azimuthally along this ring for one complete turn. The latter two steps are repeated for every consecutive ring until the aperture radius is reached. In this way we exploit the finite probe size with Gaussian shape and the milling characteristics of the FIB to generate the desired profile. In our experiments, small FIB currents (90 to 260 pA), small dwell times (≤100 µs) and multiple repetitions of the whole FIB routine yielded the best results (Figure 4b). By iteratively increasing the number of repetitions of the whole milling pattern, we determined the maximum number of repetitions for a given film thickness before the first holes are milled in the thin film. The offset thickness *t*_0_ was minimized by this procedure and the amplitude of the sinusoidal structure *t*_a_ was maximized. Depending on the film thickness and the FIB parameters, between 25 and 50 repetitions are possible. Further optimization of the pattern included an increase of the dwell time for the central point by a factor of five to achieve a similar depth as in the rings. Additionally, an offset angle between each repetition was implemented because the beam is not blanked when it moves radially outward between the rings. Without the offset angle an unwanted radial line is patterned in the PM after a few iterations. Before starting a pattern we waited for a few minutes to reduce stage drift. The total milling duration was kept below 15 min to further minimize stage-drift artifacts.

As the floated aC thin films were generally smoother compared to the FIB-prepared thin films, higher quality PM gratings could be fabricated. For the highest spatial frequency of *k*_ρ_ = 10 µm^−1^ only the floated aC thin films showed good results (cf. Figure 4a,b). To study the depth profile of the PMs, cross-section TEM lamellas were prepared by FIB milling. Bright-field TEM imaging (Figure 4c) reveals the desired sinusoidal thickness profile. However, a large offset thickness (*t*_0_ = 67 nm) and a comparably small amplitude thickness (*t*_a_ = 36 nm) are visible. This observation is unexpected because a few more repetitions lead to the formation of holes in the film, which let us expect an offset thickness in the range of only a few nanometers. The fast generation of holes may be associated with the implantation depth of the impinging Ga ions (Figure 4d). When the film becomes thin enough for Ga ions to penetrate through, there is a sudden increase in sputter yield due to additional sputtering from the PM back side. At this point, fine control over the milling process is lost due to the increased sputter yield. Accordingly, the offset thickness can only be controllably reduced to a minimum thickness that lies in the range of the penetration depth of the Ga ions in the material of the thin film. The thickness amplitude is smaller than expected, because even though the FIB is only scanned azimuthally along the minima, sputtering also takes place near the maxima of the sinusoidal structure and decreases the total thickness of the thin film with each repetition. Finer FIB probes at smaller FIB currents could improve this at the cost of increasing milling duration and possible artifacts due to stage drift. Furthermore, implanted Ga induces a dark contrast and alters the effective MIP compared to pristine aC. Since the FIB was only positioned in the minima of the sinusoidal structure, the Ga content there is higher in comparison to the maxima. Ga implantation limits fine control over the spatially defined phase shift of a PM as it is not homogeneously distributed along the PM.

The thickness patterns for VBs given by Equation 4 and Equation 5 are more complex than the concentric ring pattern for the on-axis BB. Hence, a more common approach for structuring was chosen by using bitmap files. For this purpose, grayscale bitmap files (8 bit) that mimic the thickness patterns were calculated. These can then be imported in the microscope software of the FIB system, which generates the milling pattern by calculating the dwell times based on the pixel values in the bitmap. The maximum dwell time value corresponding to a pixel value of 255 can be specified in the software and the gray values between 0 and 255 are scaled linearly. We used a maximum dwell time of 10 µs and a current of 90 pA for an aperture size of *D* = 20 µm. The depth is again controlled by adjusting the number of repetitions of the whole pattern.

**Figure 5:**
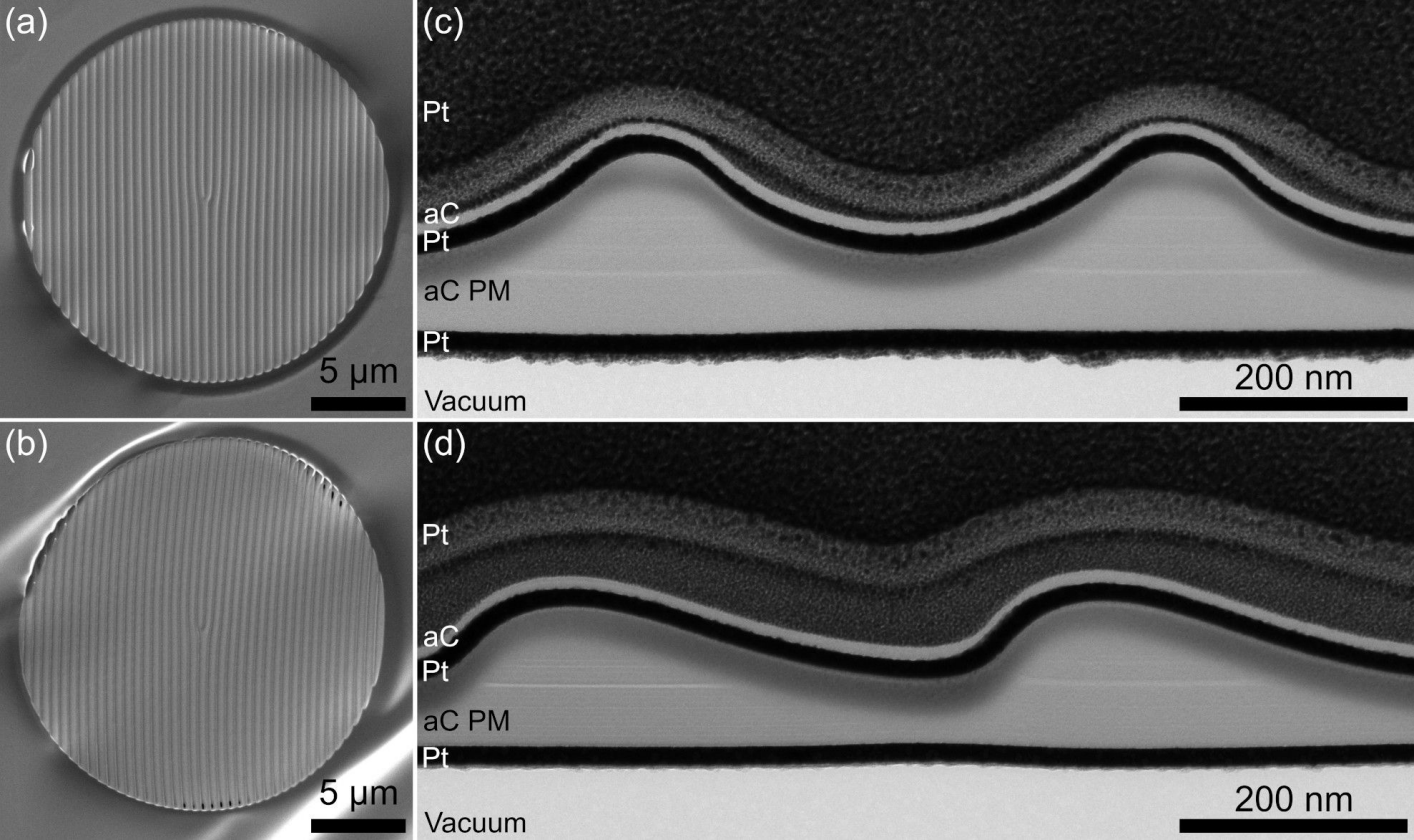
Top-view SEM images of (a) a sinusoidal and (b) a saw-tooth-shaped holographic PM for the generation of VBs. The aC thin films in both images show slight bulging. In (b) holes near the PM edge are visible. Bright-field TEM images of cross-section samples from the (c) sinusoidal and (d) saw-tooth shaped thickness profiles reveal the thickness offset and implanted Ga similar to Figure 4c.

Figure 5a and Figure 5b show the final structure for a sine and a saw-tooth holographic PM, respectively, with a diameter of 20 µm for off-axis VBs. The structural differences are only vaguely visible in the SEM micrographs. In both cases the nominal spatial frequency of the bitmaps was *f* = 2 µm^−1^ and the order *l*_0_ = 1. The experimental structure has a slightly larger *f* value, because the pattern was squeezed to a smaller diameter in order to create a small gap between the structure and the aperture edge. This choice was made because we noticed that holes preferentially form at the edge (Figure 5b) due to a smaller effective thickness by film sagging near the aperture edge and redeposition effects (see Figure 3c,d). The gap is visible as a dark ring around the patterned structure in Figure 5a,b. Slight bulging of the aC film is also visible at the aperture edges.

Cross-section samples were again prepared by FIB milling and investigated by bright-field TEM (Figure 5c,d). This time, the PMs were embedded between two sputtered Pt layers to preserve the original structure during TEM lamella preparation. Similar to the PM for the BB (Figure 4c), both cross-sections show an inhomogeneous distribution of implanted Ga (dark contrast), which is more pronounced in thinner regions due to the longer FIB dwell time. The offset thickness lies between 55 nm and 66 nm similar to the BB PM. However, the amplitude thickness is larger due to the lower spatial frequency of the structure and due to an overall increased aC thin film thickness. The sine structure was recreated rather well, whereas the saw-tooth structure shows larger deviations from the desired form because the ideally sharp edges are significantly rounded due to the finite diameter of the ion beam.

### Application of phase masks

#### Bessel beam phase mask in object plane

**Figure 6:**
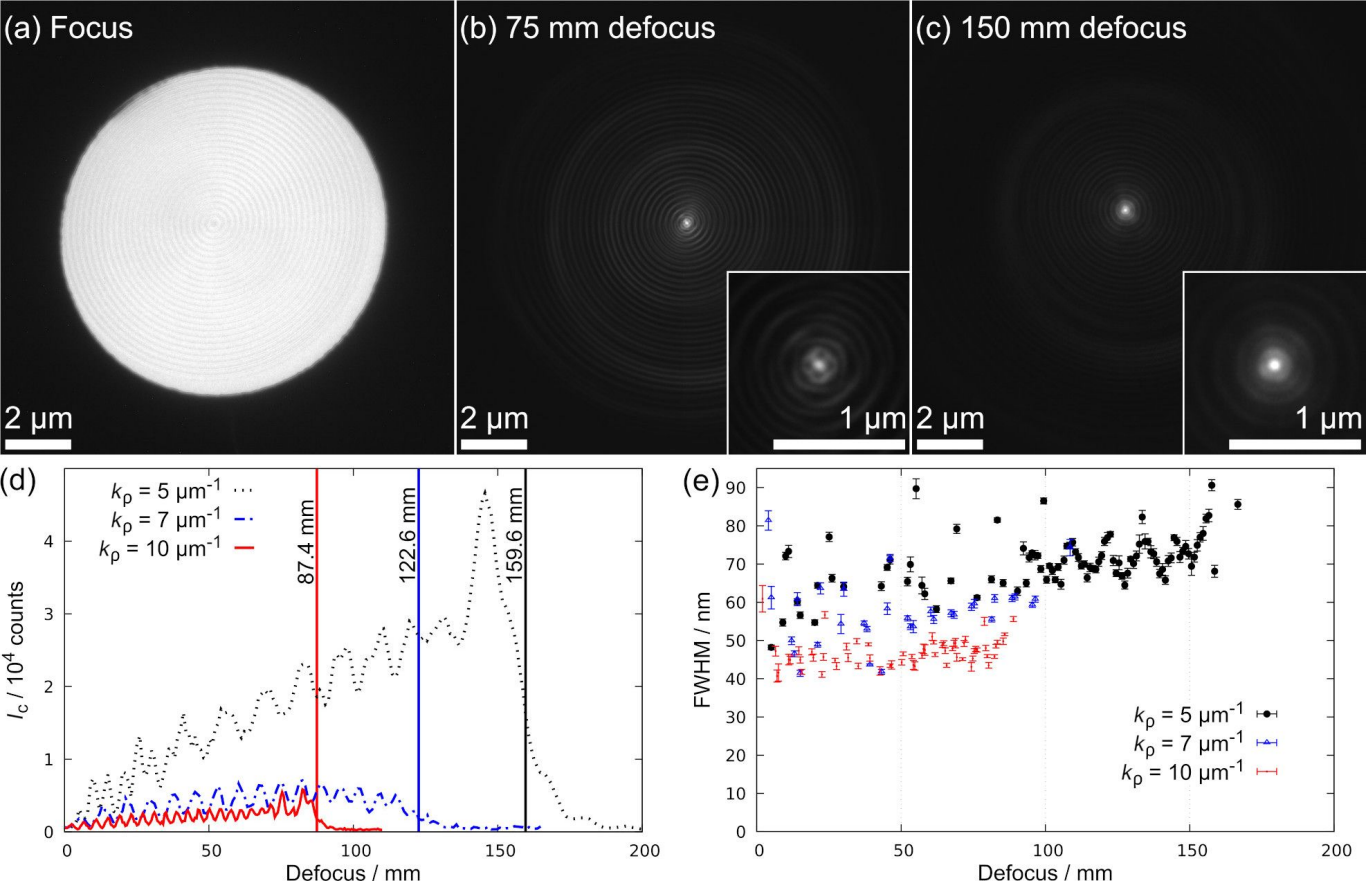
TEM images in LM mode of a PM placed in the object plane are shown in (a) to (c). (a) The image of the focused PM (*k*_ρ_ = 5 µm^−1^) shows faint amplitude contrast. Defocusing the diffraction lens to (b) 75 mm and (c) 150 mm displays the preserved shape of a BB upon propagation. The intensity in (b) and (c) is shown on a logarithmic scale to visualize the bright center and the outer rings. The inserts display the center of the respective image. Since astigmatism was corrected for the planes shown in (a) and (c) the intermediate region in (b) shows slight astigmatism. (d) The measured intensity in the central peak is plotted against the nominal defocus. Vertical lines mark the measured values for *z*_max_ for PMs with different *k*_ρ_. (e) FWHM of central maximum as a function of defocus for PMs with different *k*_ρ_. Error bars correspond to the fitting error of the Gaussian function.

PMs were first investigated as conventional samples in the object plane of a TEM which allows for a detailed analysis of a PM before placing it in the condenser system. We used a TITAN 80-300 (Thermo Fisher Scientific) operated at 300 kV (λ = 1.97 pm) equipped with a field-emission gun. Nearly parallel illumination of the PM was achieved by working in the low-magnification mode (LM mode). In this setup, the objective lens is only weakly excited (around 4%) and the diffraction lens is used for focusing. Stepwise defocusing of this lens and simultaneous image acquisition with a Gatan UltraScan camera controlled by a DigitalMicrograph script was used to trace the intensity profile of a BB upon propagation. As an example, three images of such a defocus series are shown in Figure 6a–c. The focused image of a PM in Figure 6a shows minor amplitude contrast revealing the thickness grating and the fact that PMs are not ideal phase objects. We observed astigmatism in some of the images (see, e.g., insert in Figure 6b) because we corrected the astigmatism only in two planes with the available stigmator coils (diffraction and objective stigmators) before starting the image-acquisition script. In this case, the focused PM (zero defocus, Figure 6a) and the plane with maximum intensity *I*_C_ (near *z*_max_, Figure 6c) were corrected. Therefore, astigmatism is more pronounced in the intermediate defocus region (Figure 6b). Videos of complete image series illustrating beam propagation are supplied in Supporting Information Files 1–3.

The intensity in the central maximum *I*_C_ was evaluated in dependence of the nominal defocus value given by the TEM software. Figure 6d shows measured *I*_C_ values for PMs with different *k*_ρ_ and a diameter of 10 µm. Oscillations and a linear increase of *I*_C_ are visible for all three curves in agreement with other studies [5,24]. These features are introduced due to the PM aperture and are characteristic for truncated BBs [24,31]. For larger *k*_ρ_ the astigmatically corrected planes are less separated, leading to less astigmatism and better resolved oscillations. Our measurements show that the characteristic transverse intensity profile in form of a squared Bessel function is preserved up to a maximum propagation distance and decays rapidly afterwards. This diffraction-free distance *z*_max_ (here marked with vertical lines) decreases with increasing *k*_ρ_ in agreement with Equation 2. However, the measured *z*_max_ values are only about one third of the expected value given by Equation 2, e.g., the measured value of 159.6 mm for *k*_ρ_ = 5 µm^−1^ is only about 31% of the calculated diffraction-free distance of 507.6 mm. This may be caused by the focusing effect of the lenses that decreases the maximum propagation distance. Also, the nominal defocus value may not correspond to the actual propagation distance due to faulty calibration. A larger number of concentric rings was observed for larger *k*_ρ_ values as expected from theory.

Another aspect is, that the overall intensity decreases more rapidly than expected for larger *k*_ρ_. This effect could result from an insufficient amplitude thickness of the sinusoidal pattern. Due to the limited resolution of the FIB, finer structures are not milled with the desired depth resulting in an unfavorable phase shift and consequently less efficiently generated BBs. For comparison, free propagation of BBs for the same experimental parameters is simulated in Figure S1 in Supporting Information File 1. Overall, a decrease in size of the central maximum was observed with increasing *k*_ρ_ (Figure 6e), although not as drastic as expected from theory. For example, doubling of *k*_ρ_ does not generate a central maximum with half diameter. The plotted values were measured by fitting a Gaussian function to the central lobe and evaluating the full width at half maximum (FWHM). As the beam intensity is oscillating upon propagation, the FWHM of the central maximum also varies significantly, which leads to a spread in the values instead of a constant central probe size.

#### Properties of generated Bessel beams

For applications of a BB as a STEM probe the non-diffracting beam shape must be obtained in the object plane where the sample is located. This is achieved by placing a PM in the condenser system of a TEM. We used a Philips CM200 FEG/ST operated at 200 kV and positioned a PM with *k*_ρ_ = 10 µm^−1^ and a diameter of 10 µm in the plane of the second condenser (C2) aperture. Illumination of the PM can be controlled via excitation of the first condenser lens (C1), which is accomplished in this microscope by changing the value of the so-called “spot size”. The imaging optics of the microscope was set to conventional TEM where the objective lens focuses on the object plane. No sample was inserted in the object plane for the following measurements to investigate the intrinsic behavior of the generated BB.

The strength of the C2 lens was altered to control the properties of the BB. Its excitation determines the demagnification and actual shape of the generated BB in the object plane, meaning that one particular effective propagation distance after propagation through the PM is focused onto the object plane. For an intense probe one may, e.g., choose a propagation distance with a pronounced central peak and the largest *I*_C_ value according to the characteristic curves shown in Figure 6d.

**Figure 7:**
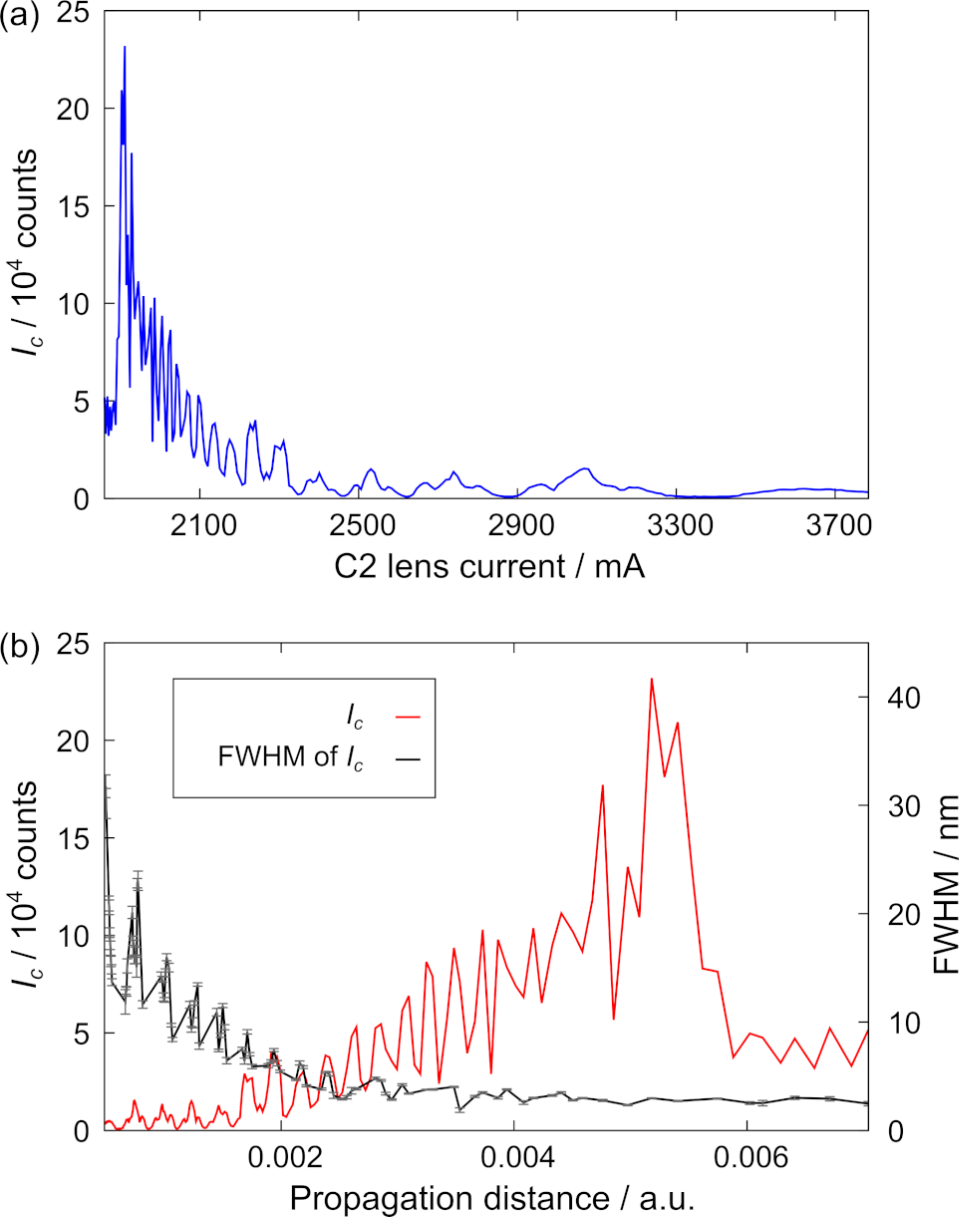
(a) Intensity in the center of a BB *I*_C_ plotted as a function of the C2 lens current. The curve is reversed and compressed compared to the curves in Figure 6d. (b) Rescaling with Equation 6 linearizes the abscissa and qualitatively shows the propagation distance. Oscillations and the linear increase in *I*_C_ confirms the successful generation of a BB in the object plane. The FWHM of the central peak decreases upon propagation as a change of C2 excitation also changes the demagnification. Error bars correspond to the fitting error of the Gaussian function.

The beam profile upon propagation was traced by systematically changing the C2 excitation. As the image acquisition was performed manually and not via a script we corrected astigmatism for every image. Afterwards, the central intensity *I*_C_ was analyzed in dependence of the C2 current (Figure 7a). Compared to the plot in Figure 6d, the curve is mirrored and compressed for smaller currents, which is due to a non-linear change in focal strength with increasing lens current. To link the C2 currents to an effective propagation distance *d*_eff_ the following equations

[6] $$d_{\text{eff}}=\frac{d_{\text{phys}}}{D\left(I_{\text{C2}}\right)}\;\;\text{with}\;\;D\left(I_{\text{C2}}\right)=v\left(I_{\text{C2}}-I_{\text{C2}\text{,BFP}}\right)$$

were used. The physical propagation distance *d*_phys_ is demagnified by a factor *D* depending on the C2 lens current *I*_C2_. The variable *I*_C2,BFP_ denotes the C2 lens current at which the back focal plane of the C2 lens coincides with the object plane. The factor *v* relates *D* with *I*_C2_ and can be determined by a reference measurement with an object of known physical size *d*_phys_. Even though in our case the real propagation distance is unknown, the *I*_C_ values can still be qualitatively plotted against the propagation distance with Equation 6, *d*_phys_ = *v* = 1 and *I*_C2,BFP_ = 1718 mA (Figure 7b). Again, oscillations and an increase in the central intensity are observed and demonstrate the successful generation of a BB in the object plane.

Additionally, the diameter of the central maximum was evaluated by fitting a Gaussian function to the intensity profile and evaluating the FWHM. The data is shown in Figure 7b with the corresponding values of *I*_C_. If the C2 excitation is varied to trace the intensity profile, the demagnification also changes. Indeed, the central beam size decreases with increasing effective propagation distance due to the stronger lens demagnification. This observation means that a larger *z*_max_ (smaller *k*_ρ_, see Equation 2) leads to a smaller relative probe size. However, a smaller *k*_ρ_ simultaneously results in a broader central peak as discussed earlier (Figure 6e), which requires a compromise between the two conditions in order to minimize the FWHM. For the most intense *I*_C_ value, a FWHM of (3.00 ± 0.03) nm is found. Another important aspect is that the relative intensity of the central peak is reduced with increasing number of concentric rings, making a large *k*_ρ_ value not suitable if only the central maximum is intended to be used as an electron probe. In a related publication we described a way towards a possible application of BBs by combining lower spatial frequencies with a higher demagnification of the condenser lens system [25].

#### Properties of generated vortex beams

**Figure 8:**
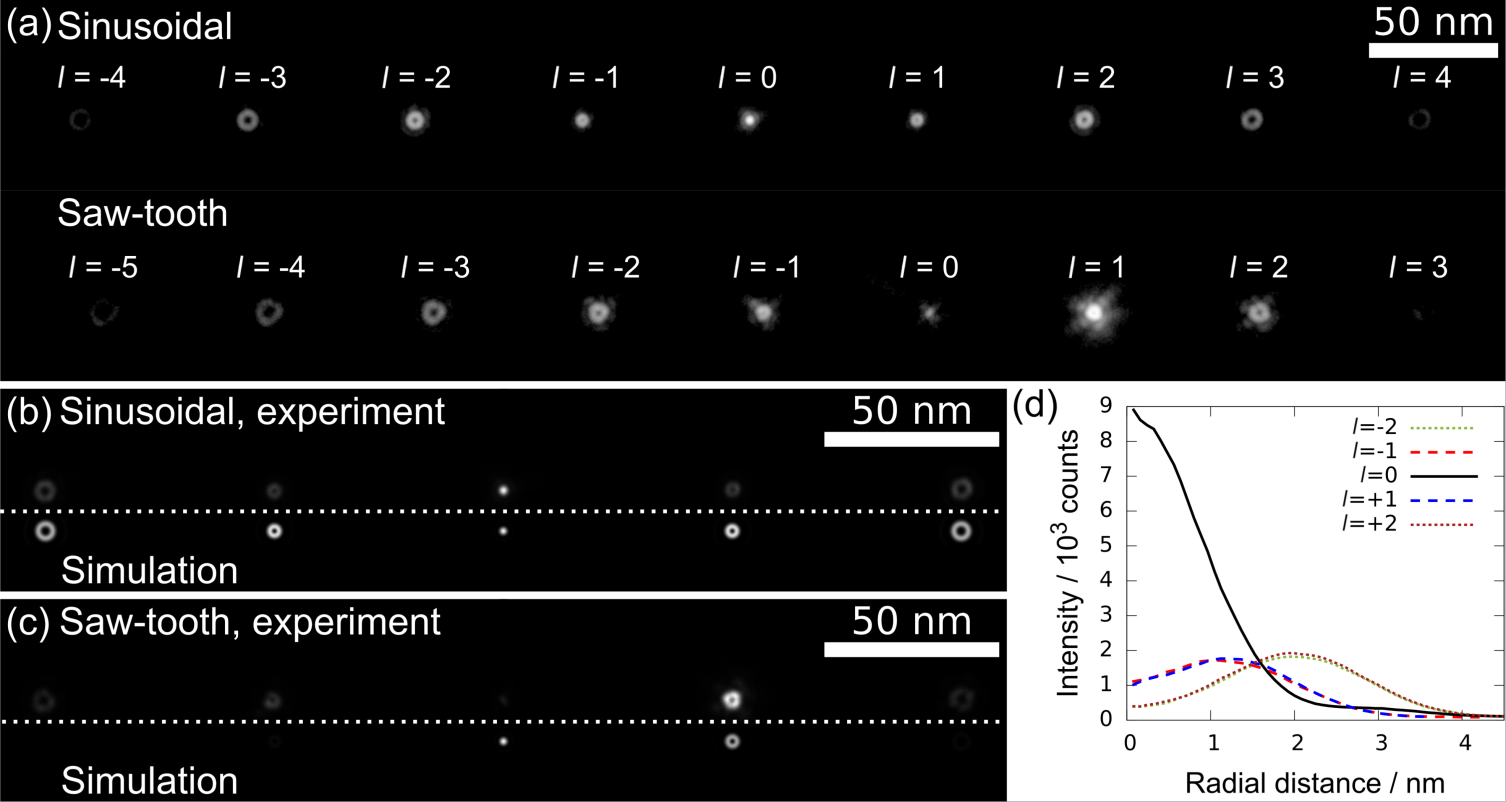
(a) Generated VBs in the object plane of a Philips CM200 for the sinusoidal and saw-tooth pattern, respectively. The images are displayed in logarithmic intensity scale to visualize multiple diffraction orders with strongly varying intensity. The size of the doughnut-shaped beam profile increases with the topological charge *l*. Magnified sections for *l* = −2 to *l* = 2 for (b) sinusoidal and (c) saw-tooth patterns with linear intensity scale are compared with simulations. (b) The sinusoidal pattern shows good agreement, whereas for (c) a larger deviation is observed. (d) The azimuthally averaged intensities of the measured beams in (b) are plotted against the radial distance from the center of each beam.

The holographic PMs shown in Figure 5a and Figure 5b were placed in the C2 aperture position to generate VBs in the object plane of a Philips CM200 FEG/ST. The strength of the C2 lens was adjusted to position the back focal plane of the C2 lens in the object plane which is imaged by the objective lens (Figure 8a). The different beam orders are well separated and the diameter of the expected doughnut-shaped VBs increases with topological charge *l*. Depending on the fine structure of the thickness pattern a symmetric or asymmetric intensity distribution around the *l* = 0 order beam is visible as expected [28]. Indeed, much of the total intensity is concentrated in the *l* = 1 beam for the saw-tooth shaped PM.

Consistency with theory was examined by accompanying simulations with a self-written MATLAB program for the first few diffraction orders for sinusoidal and saw-tooth shaped thickness patterns (Figure 8b,c). As the displayed VBs form in the far-field Fraunhofer regime, the simulated images were calculated by Fourier transformation of an electron wave that acquires a phase shift according to an ideal sinusoidal or saw-tooth shaped phase mask. Critical simulation parameters were taken from the experiment. For example, the focal length for the C2 lens was calculated from the spacing of the diffracted beams and the amplitude and offset thickness were taken from the TEM images (Figure 5c,d), respectively. The sinusoidal-shaped pattern shows good agreement with the simulation (Figure 8b), whereas a larger deviation is observable for the saw-tooth pattern (Figure 8c). The simulated ideal saw-tooth pattern shows a higher concentration of beam intensity than the experiment in the beams of order *l* = 0 and *l* = 1, and nearly no intensity in the other orders. The experimental saw-tooth PM generates a weak beam of order *l* = 0 and more pronounced beams of order *l* = −2, *l* = −1 and *l* = 2. These discrepancies can be attributed to the noticeable deviation from the ideal saw-tooth thickness pattern and the simple simulation model used (see also Supporting Information File 1, Figure S2).

In Figure 8d the azimuthally averaged experimental intensities of the diffracted beams shown in Figure 8b are plotted against the radial distance from the center of each diffraction order. The graph shows the highly symmetric intensity spread resulting from the sinusoidal PM as the curves for corresponding orders (e.g., *l* = −1 and 1) agree very well. Again, an increase in beam width for larger *l* is visible. The intensity in the center of the doughnut shape for the *l* ≠ 0 beams does not reach zero because of limited spatial coherence of the electron beam in combination with lens aberrations [32].

## Conclusion

In conclusion, we have shown that amorphous carbon (aC) can be used as an alternative material to commonly used Si*_x_*N*_y_* for beam-shaping phase masks (PMs). The most challenging aspect is the fabrication of smooth aC thin films in combination with an aperture. Floated aC thin films from mica yielded best results, although possible delamination of the film makes these PMs in general less robust compared to Si*_x_*N*_y_*. No qualitative degradation of beam shape quality was observed during PM application, which suggests that no charging or contamination was present.

Bessel (BBs) and vortex beams (VBs) were successfully generated in transmission electron microscopes. For BBs, we observed that higher spatial frequencies in the thickness grating of the PM reduce the size of the central maximum. The decrease in diameter comes at the expense of an increasing number of unwanted concentric rings. Moreover, the achievable demagnification caused by the lenses decreases due to smaller *z*_max_. This makes PMs with smaller spatial frequencies in the concentric ring pattern more promising for the formation of small electron probes with high depth of focus, which is desirable for applications. The generated VBs showed the expected behavior reported by other groups, although the saw-tooth shaped thickness profile showed considerable deviations from the ideal structure.

## Experimental

For (positive) optical lithography we used a spin coater from POLOS to coat our wafers with a TI35ES resist (Microchemicals GmbH). Lithography masks for a mask aligner (MA6 by SÜSS MicroTec, light source with λ = 356 nm) were fabricated with a DWL66 laser lithography system (Heidelberg Instruments). After illumination of the unmasked regions, the resist was developed in AZ 726 MIF (Microchemicals GmbH). To deposit Cr, Pt and aC, electron-beam physical vapor deposition (PVD, PVD75 by Kurt J. Lesker Company) was used. Apertures and thickness profiles were structured with the Ga ion beam of a Helios G4 FX SEM/FIB dual-beam instrument (Thermo Fischer Scientific). The Ga ion energy was set to 30 keV in all applications.

## Supporting Information

Supporting Information Files 1 to 3 contain videos of BB propagation corresponding to the intensity curves shown in Figure 6d. The intensity in Supporting Information File 2 and Supporting Information File 3 is normalized with respect to the maximum intensity in the corresponding image series. The contrast in Supporting Information File 4 is modified to enhance visibility. Image dimensions are 2.7 µm by 2.7 µm. The playback speed of Supporting Information File 4 and Supporting Information File 2 corresponds to 7 mm defocus per second. The playback speed of Supporting Information File 3 corresponds to 3.5 mm defocus per second. Supporting Information File 1 contains accompanying simulations for Figure 6 and Figure 8.

File 1 BB propagation for *k*_ρ_ = 5 µm^−1^.

File 2 BB propagation for *k*_ρ_ = 7 µm^−1^.

File 3 BB propagation for *k*_ρ_ = 10 µm^−1^.

File 4 Accompanying simulations for Figure 6 and Figure 8.
