# Supplementary material for: Fabrication of phase masks from amorphous carbon thin films for electron-beam shaping
Source: Beilstein J Nanotechnol. 2019 Jun 25;10:1290–302. doi: 10.3762/bjnano.10.128 (PMC6604735; doi:10.3762/bjnano.10.128)
Supplement: File 4 — Accompanying simulations for Figure 6 and Figure 8. [file Beilstein_J_Nanotechnol-10-1290-s004.pdf]

BEILSTEIN JOURNAL OF NANOTECHNOLOGY

Supporting Information

for

Fabrication of phase masks from amorphous carbon thin films for electron-beam shaping

Lukas Grünewald, Dagmar Gerthsen and Simon Hettler

Beilstein J. Nanotechnol. **2019,** *10,* 1290–1302. doi:10.3762/bjnano.10.128

Accompanying simulations for Figure 6 and Figure 8

License and Terms: This is a supporting information file under the terms of the Creative Commons Attribution License (http://creativecommons.org/licenses/by/4.0). Please note that the reuse, redistribution and reproduction in particular requires that the authors and source are credited.
The license is subject to the *Beilstein Journal of Nanotechnology* terms and conditions: (https://www.beilstein-journals.org/bjnano)

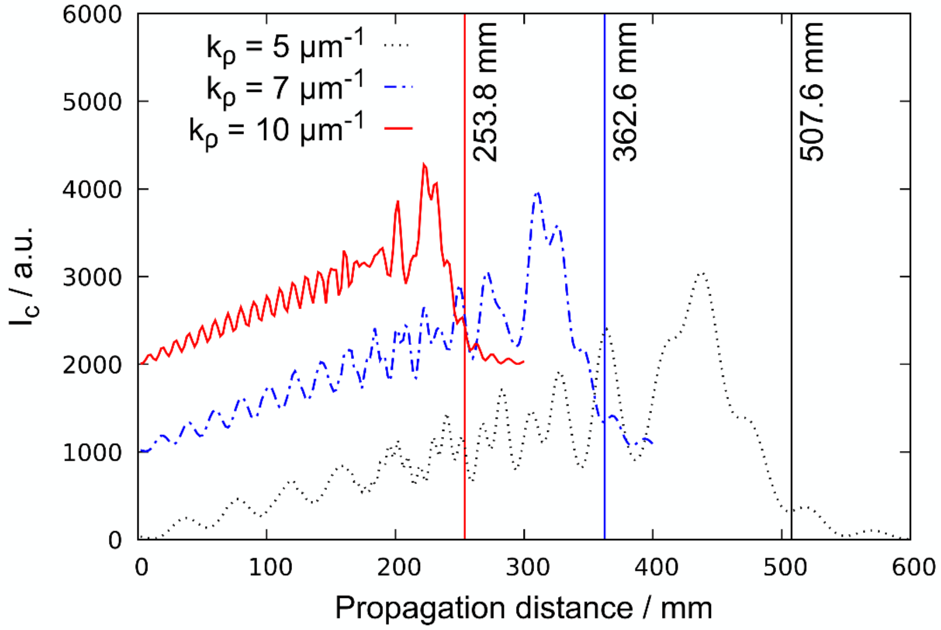

Figure S1: Simulation of the intensity in the center of a Bessel beam (BB) I_c upon propagation behind a beam-shaping phase mask (PM). The curves were simulated for a PM diameter $D = 10\ \mu\mathrm{m}$, $\lambda = 1.97\ \mathrm{pm}$ and different k_ρ according to the experimental measurements shown in Figure 6. The curves for $k_\rho = 7\ \mu\mathrm{m}^{-1}$ ($k_\rho = 10\ \mu\mathrm{m}^{-1}$) are offset vertically by 1000 (2000), respectively, for better visibility. The maximum propagation distances $z_{\max}$ were calculated with Equation 2 and are marked with vertical lines.

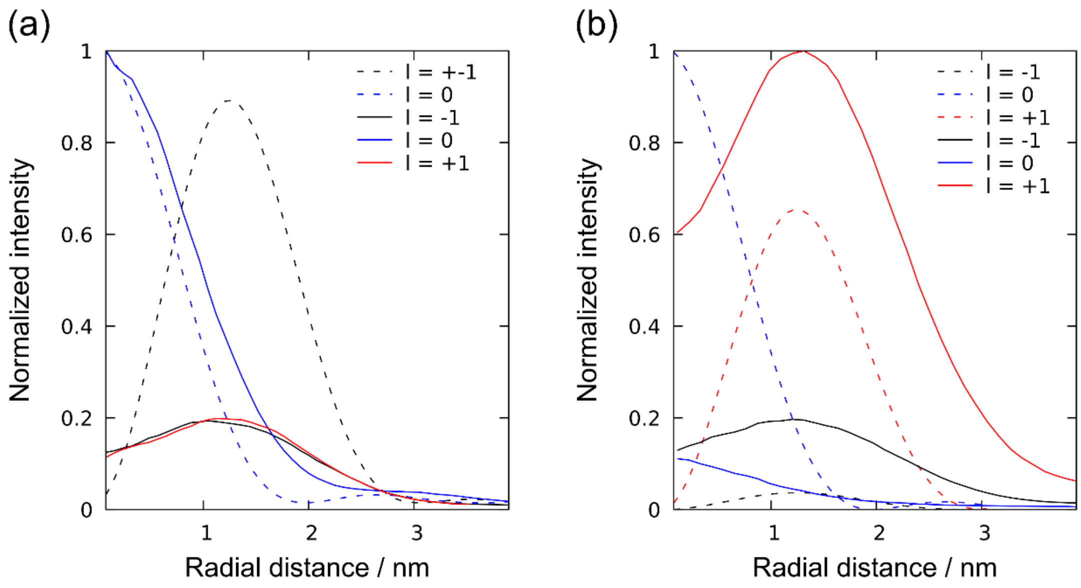

Figure S2: Comparison between simulated (dotted lines) and measured (solid lines) azimuthally averaged intensities of vortex beams generated by (a) sinusoidal and (b) saw-tooth shaped thickness patterns. The topological charge is denoted by l. We used a simple simulation model based on a perfect PM thickness profile given by Equation 4 and Equation 5. Values for $t_0 = 64\ \mathrm{nm}$ $(t_0 = 55\ \mathrm{nm})$ and $t_\mathrm{a} = 69\ \mathrm{nm}$ $(t_\mathrm{a} = 59\ \mathrm{nm})$ for the sinusoidal (saw-tooth shaped) PM were taken from the cross-section image shown in Figure 5c (Figure 5d). The resulting phase shift was calculated for a PM of pure aC with a mean inner potential of $V_\mathrm{MIP} = 9\ \mathrm{V}$ and an electron energy of 200 keV. The focal length of the focusing lens was measured to be 13 mm from the spacing between the generated vortex beam orders and the known spatial frequency of the thickness pattern. While the simulated and experimental curves do qualitatively agree, discrepancies in intensities are visible, which may be attributed to the simple simulation model used. The imperfections of the PMs (Ga implantation and non-ideal thickness profiles) and other aspects of the microscope (incoherence and lens aberrations) must be taken into account for a better description of the measured intensity distribution.
